# Interactive plant growth regulator and fertilizer application dataset on growth and yield attributes of tomato (*Solanum lycopersicum* L.)

**DOI:** 10.1016/j.dib.2024.111136

**Published:** 2024-11-14

**Authors:** Joydeb Gomasta, Jahidul Hassan, Hasina Sultana, Emrul Kayesh

**Affiliations:** Department of Horticulture, Bangabandhu Sheikh Mujibur Rahman Agricultural University, Gazipur 1706, Bangladesh

**Keywords:** Gibberellic acid, Salicylic acid, Inorganic fertilizer, Vegetative growth, Flowering and fruiting, Yield, Vegetables, Fluctuating weather

## Abstract

Tomato is known for its remarkable contents of vitamins, minerals and antioxidants. A pot experiment was performed during the winter-summer transition from December 2022 to April 2023 combining low to high fertilizer rates and plant growth regulators (PGRs). The objective was to decrease the utilization of artificial fertilizers through the application of PGRs. Besides recommended dose (12 g of urea, 10 g of TSP, 5 g of MoP, 3 g of Gypsum, 0.5 g of ZnSO_4_, and 0.5 g of Boric acid per plant), the experiment involved applying fertilizers at 80 %, 90 %, and 110 % of the recommendation plus a control (farmers practice). Furthermore, PGRs including gibberellic acid (GA_3_), naphthalene acetic acid (NAA), 4-chlorophenoxy acetic acid (4-CPA) and salicylic acid (SA) were applied at a concentration of 50 ppm. Setting the experiment in a factorial randomized complete block design (RCBD), data on vegetative and reproductive plant behaviors were registered to assess the interactive influence of inorganic nutrients and PGRs on tomato growth and development. The dataset obtained from the experiment focuses on how plant growth regulators like GA_3_ and SA significantly ameliorated the reduced chemical fertilizer induced nutrient deficit. Plants had superior growth and yield with GA_3_ and SA applications, whereas NAA and 4-CPA accounted for inferior crop health and production even lower than that of control (no PGRs). In addition, as a function of PGR treatment, the tomato plants showed no distinguishable variations in vegetative and reproductive behaviors for fertilizer doses from 80 % to 110 % of recommendation. The dataset, thus, can encourage the farmers, researchers and policymakers for sustainable tomato cultivation with minimal inorganic fertilization through incorporating judicious PGRs. Future studies should focus on cellular and metabolic changes in tomato after PGR-fertilizer interactive use.

Specifications Table

**Table:** 

Subject	Agricultural Science: Horticulture, Agronomy and Crop Science
Specific subject area	Vegetable cultivation, Plant Growth Regulation, Horticultural Production
Type of data	Tables and Figures
Data collection	A pot experiment was conducted during the 2022–2023 season (December 2022 to April 2023) with tomato by incorporating lower to higher fertilizer rates than recommendation and different plant growth regulators (PGRs). BARI Tomato-14 (a variety released from Bangladesh Agricultural Research Institute) was used as planting material. Fertilizer doses like 100 % (12 g of urea, 10 g of TSP, 5 g of MoP, 3 g of Gypsum, 0.5 g of ZnSO_4_, and 0.5 g of Boric acid per plant), 80 %, 90 % and 110 % of recommendation were used besides control (farmers’ practice) for factor one. Whereas factor two comprised of PGRs namely gibberellic acid (GA_3_), naphthalene acetic acid (NAA), 4-chlorophenoxy acetic acid (4-CPA), and salicylic acid (SA) at 50 ppm concentration. Data were recorded on plant height (cm), base diameter (mm), number of branch and leaves per plant, number of leaflets per leaf, individual leaf area (cm^2^), canopy dimension (cm), SPAD value, days to flowering, number flower cluster per plant, number of flowers and fruits per cluster, total number of flowers and fruits per plant, fruit set percentage, single fruit weight (g) and fruit yield per plant (kg).
Data source location	The research plot was situated at the Horticulture research field at the Department of Horticulture, Bangabandhu Sheikh Mujibur Rahman Agricultural University under Madhupur Tract (Agro-ecological Zone 28) of Bangladesh. Geographically the field had its location at 24.037°N latitude and 90.398°E longitude (8.4 m elevation from the mean sea level).
Data accessibility	Repository name: Mendeley Data Data identification number: https://doi.org/10.17632/jnm4z2vmfj.1 Direct URL to data: https://data.mendeley.com/datasets/jnm4z2vmfj/1
Related research article	Not applicable

## 1 Value of the Data

• The dataset of the experiment indicated that PGRs can significantly influence the nutrient regulation for sustainable growth and yield of tomatoes under fluctuating winter atmospheres in the sub-tropical regions. Farmers, entrepreneurs and researchers will get a comprehensive note on how specific PGRs can augment or diminish the growth and yield of tomato grown with varied fertilizer doses at field conditions. The adverse as well as beneficial effects observed with these PGRs highlight the importance of careful selection and application of PGRs to achieve desired outcomes in crop production.
• From these observations, farmers can minimize the excess fertilization for tomato cultivation by complementing plant growth regulators namely GA_3_ and SA. The outcomes of this study could provide valuable insights into sustainable agricultural practices and contribute to the development of environmentally friendly cultivation techniques that align with the global goals of reducing agricultural pollution and enhancing food security. This research will build on existing knowledge and address the critical need for innovative solutions in modern agriculture, offering practical benefits to farmers and promoting ecological balance in tomato production systems.
• The dataset can be used by researchers to validate existing theories or formulate new hypotheses regarding the application of plant growth regulators (PGRs) in horticulture, specifically in tomato cultivation. This data offers a valuable basis for further investigations into the combined effects of PGRs and mineral fertilizers on different crops.
• Entrepreneurs and policymakers can utilize these findings to develop guidelines and recommendations for the use of PGR-based fertilizers in vegetable cultivation, promoting sustainable and profitable agricultural practices. Establishing safe and effective standards for the use of GA_3_ and SA in horticulture is essential to ensure consumer and environmental safety.
• Above all, the dataset plays a crucial role in improving the efficiency of tomato production, enhancing the economic viability and sustainability of the vegetable farming industry. It provides a scientific basis for optimizing the use of plant growth regulators to reduce the reliance on chemical fertilizers, ensuring better outcomes for all stakeholders involved in the tomato production chain.

## 2 Background

Tomatoes (*Solanum lycopersicum*) are one of the most widely cultivated and consumed vegetables globally, valued for their nutritional content and versatility in culinary uses. The fruit vegetable has high contents of carotenoids, representing the main source of lycopene in the human diet [1]. Tomatoes also have the naturally occurring antioxidants vitamins C and E [2] as well as large amounts of metabolites, such as sucrose, hexoses, citrate, malate and ascorbic acid [3] to promote human health. Globally, 251.69 million tons of tomato has been produced from 6.16 million hectares of land [4], while Bangladesh grew 4.70 lakh tons from 0.31 lakh hectares of land during 2022–23 [5]. This production has proved short to meet the country's overall daily vegetable requirement of 235 g per person. Therefore, the growers are encouraged to increase the total production, but the only thing farmers are used to practice for increasing the yield and production is the excessive and indiscriminate use of inorganic fertilizers neglecting the Fertilizer Recommendation Guide [6]. Such practice can lead to environmental degradation, soil nutrient imbalances, and increased production costs [7]. Though judicious increase in fertilizer use can improve yield and soil health [8,9], the excessive use of chemical fertilizers contributes to the emission of greenhouse gases, water pollution through runoff, and long-term soil fertility decline due to nutrient leaching and altered microbial communities [10]. In addition, continuous and steady application of inorganic fertilizers leads plant tissues to frequently absorb and accumulate heavy metals, which consequently decreases the nutritional and grain quality of crops [11]. Therefore, there has been an increasing need to explore alternative strategies that can reduce dependency on chemical fertilizers while maintaining or enhancing crop productivity.

One promising approach is the use of plant growth regulators (PGRs), which are organic compounds that, in small concentrations, can influence various physiological processes in plants, such as cell division, elongation, flowering, and fruit development. PGRs like gibberellic acid (GA_3_), naphthalene acetic acid (NAA), and salicylic acid (SA) have shown potential in enhancing plant growth, yield and stress tolerance [12,13]. Previous studies have indicated that PGRs can improve nutrient uptake efficiency, increase photosynthetic rates, and enhance stress resilience, thereby potentially reducing the need for chemical fertilizers [14,15]. By optimizing the use of PGRs, it may be possible to achieve a more sustainable tomato production system that minimizes environmental impact and promotes healthier soil ecosystems.

However, researchers worldwide optimized fertilizer dose [16,17] as well as PGR concentrations [18,19] to enhance yield of tomato at field conditions. Nonetheless, rigorous research reports are meagre on PGR-fertilizer interactions, how growth regulators can work for deficient inorganic nutrient inputs to obtain superior yield in tomato. Considering all, the proposed study aimed at investigating the effectiveness of specific PGRs in reducing chemical fertilizer requirements for tomato cultivation in field conditions.

## 3 Data Description

The current dataset demonstrates the phenomena of how regulated use of plant growth regulators can modulate the overuse of inorganic fertilizers for tomato cultivation under fluctuating weather conditions during late winter. Interactive application of plant growth regulators and mineral fertilizers displays that the five PGRs (control, and GA_3_, SA, NAA and 4-CPA at 50 ppm), five fertilizer doses (control, 80 %, 90 %, 100 % and 110 % of recommendation) and their interactions had significant (*p* < 0.05) influences on the vegetative and the reproductive behaviors of tomato (Table 1).

**Table 1:** ANOVA table for mean square values of growth and yield contributing attributes of tomato after interactive fertilizer and plant growth regulator applications.

Sources of variation	Mean square value																		
	DF	PHT	BDM	BNN	LFN.P	LFT.L	INDL	SLFA	CNPY	SPAD	DFLR	FLC.P	FLR.C	FLR.P	FRT.C	FRT.P	FSP	FWT	FYP
Fertilizer rate (N)	4	104.684 *	0.181 **	0.607 ns	48.37 *	1.095 *	0.096 ns	955.204 *	58.361 *	14.856 ns	2.831 ns	6.752 **	0.329 ns	604.429 **	0.389 ns	289.22 **	25.135 *	39.273 *	1.961 **
Plant growth regulator (P)	4	6059.68 **	2.23 **	14.51 **	1598.703 **	38.819 **	10.236 **	22,370.117 **	2053.005 **	81.775 **	196.378 **	480.018 **	37.971 **	50,459.6 **	12.434 **	20,546.221 **	98.059 **	953.253 **	104.789 **
*N* × *P*	16	20.839 ns	0.017 ns	0.322 ns	6.101 ns	0.459 ns	0.107 ns	1243.199 **	7.82 ns	8.67 ns	0.783 ns	0.734 ns	0.269 ns	52.61 ns	0.281 ns	27.709 ns	15.974 *	4.123 ns	0.217 ns
Residual	48	31.476	0.037	0.392	13.392	0.316	0.294	347.999	18.268	14.09	8.915	0.497	0.23	74.369	0.215	50.454	8.289	11.816	0.151

Here, DF: degrees of freedom, PHT: Plant height, BDM: Base diameter, BNN: Number of branches per plant, LFN.P: Number of leaves per plant, LFT.L: Number of leaflets per leaf, INDL: Internode length, SLFA: Single leaf area, CNPY: Canopy dimension, SPAD: SPAD value, DFLR: Days to flowering, FLC.P: Number of flower clusters per plant, FLR.C: Number of flowers per cluster, FLR.P: Number of flowers per plant, FRT.C: Number of fruits per cluster, FRT.P: Number of fruits per plant, FSP: Fruit set percentage, FWT: Individual fruit weight, FYP: Fruit yield per plant; *,**: Significant at 5 % and 1 % level of probability, ns: Not-significant.

**Fig. 1:**
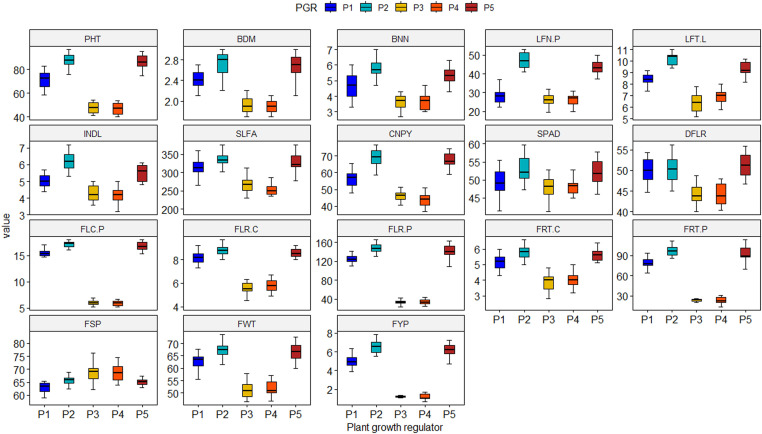
Boxplot demonstrating the main effect of plant growth regulator on vegetative growth, flowering, fruiting and yield of tomato. Here, P_1_, P_2_, P_3_, P_4_ and P_5_ denote control (no PGR), GA3, SA, NAA and 4-CPA at 50 ppm, respectively; PHT: Plant height (cm), BDM: Base diameter (cm), BNN: Number of branches per plant, LFN.P: Number of leaves per plant, LFT.L: Number of leaflets per leaf, INDL: Internode length (cm), SLFA: Single leaf area (cm^2^), CNPY: Canopy dimension (cm), SPAD: SPAD value, DFLR: Days to flowering, FLC.P: Number of flower clusters per plant, FLR.C: Number of flowers per cluster, FLR.P: Number of flowers per plant, FRT.C: Number of fruits per cluster, FRT.P: Number of fruits per plant, FSP: Fruit set percentage, FWT: Individual fruit weight (g), FYP: Fruit yield per plant (kg).

**Fig. 2:**
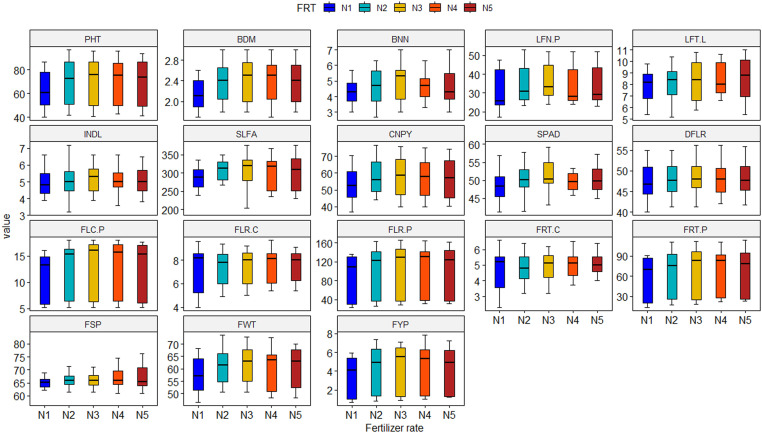
Boxplot demonstrating the main effect of fertilizer treatment on vegetative growth, flowering, fruiting and yield of tomato. Here, N_1_, N_2_, N_3_, N_4_ and N_5_ represent control (farmers’ practice), 80 %, 90 %, 100 % and 110 % of recommended fertilizer dose, respectively; PHT: Plant height (cm), BDM: Base diameter (cm), BNN: Number of branches per plant, LFN.P: Number of leaves per plant, LFT.L: Number of leaflets per leaf, INDL: Internode length (cm), SLFA: Single leaf area (cm^2^), CNPY: Canopy dimension (cm), SPAD: SPAD value, DFLR: Days to flowering, FLC.P: Number of flower clusters per plant, FLR.C: Number of flowers per cluster, FLR.P: Number of flowers per plant, FRT.C: Number of fruits per cluster, FRT.P: Number of fruits per plant, FSP: Fruit set percentage, FWT: Individual fruit weight (g), FYP: Fruit yield per plant (kg).

While considering the vegetative growth parameters for PGR treatments, GA_3_ exhibited superiority over the others for plant height (87.90 cm), base diameter (2.72 cm), number of branches (5.85 per plant), number of leaves (47.13 per plant), canopy spread (69.29 cm), internode length (6.17 cm) and Soil Plant Development Analysis (SPAD) value (53.08) of tomato. Once again, reproductive traits like number of flower clusters (16.85 per plant), number of flowers (148.12 per plant) and fruits (97.33 per plant), single fruit weight (67.53 g) and yield (6.57 kg per plant) of tomato were noticed maximum in GA_3_. The salicylic acid demonstrated statistical parity with that of GA_3_ (Fig. 1, Supplementary Table 1). On the other hand, among the fertilizer treatments, fertilizer doses 80 to 110 % exhibited statistical similar performances to induce plant growth, flowering, fruiting and yield of tomato under study. These doses differed only from the control fertilization (Fig. 2, Supplementary Table 1). Interactions revealed that GA_3_ and SA in combination with any of the fertilizer doses from 80 % to 110 % of recommendation had superiority over other combinations. Whereas NAA and 4-CPA interacted fertilizer treatments demonstrated statistical inferiority for growth and reproduction of tomato (Table 2).

**Table 2:** Interaction of plant growth regulator and fertilizer on vegetative and reproductive traits of tomato.

Treatment combination		PHT	BDM	BNN	LFN.P	LFT.L	INDL	SLFA	CNPY	SPAD	DFLR	FLC.P	FLR.C	FLR.P	FRT.C	FRT.P	FSP	FWT	FYP
P_1_	N_1_	61.97cd	2.13b-d	4.00b-f	25.20cd	8.50c-f	4.87a-e	286.40b-g	52.60d-h	49.50	49.00	13.57c	8.27a	112.17d	5.27a-f	71.47c	63.68	58.70a-d	4.18f
	N_2_	80.97ab	2.43a-d	5.23a-f	45.00a	9.67a-c	6.00a-c	321.11a-e	64.40a-d	51.77	49.67	14.90bc	8.93a	132.67a-d	5.93a	87.97a-c	66.32	65.73a	5.78a-e
	N_3_	47.90d	1.90cd	3.67d-f	23.33cd	5.93h	4.40b-e	264.40e-h	45.90f-h	45.63	43.00	5.67d	4.83b	27.46e	3.13h	17.83d	64.73	48.83d	0.88g
	N_4_	46.57d	1.83d	3.67d-f	21.00d	6.93f-h	4.03e	248.43f-h	41.43h	45.17	43.67	5.67d	5.03b	28.33e	3.27h	18.34d	64.23	49.90d	0.91g
	N_5_	79.67a-c	2.37a-d	5.13a-f	42.67ab	8.67b-f	5.30a-e	311.31a-e	62.63a-d	50.90	51.33	14.87bc	8.50a	126.57b-d	5.50a-d	81.97a-c	64.68	64.20ab	5.24c-f
P_2_	N_1_	72.00bc	2.43a-d	4.57a-f	27.67cd	8.47c-f	5.13a-e	310.94a-e	56.03c-g	47.50	50.00	15.33a-c	7.87a	120.67cd	4.87a-g	74.67bc	61.72	62.37a-c	4.66ef
	N_2_	89.93a	2.77a	5.87ab	49.00a	9.80a-c	6.53a	326.65a-d	71.27a	53.03	50.67	17.20a	8.93b	153.56ab	5.93a	101.96a	66.36	68.57a	6.98a
	N_3_	48.43d	2.00cd	3.47ef	26.00cd	6.13gh	4.23de	282.99c-h	47.77e-h	49.33	44.67	6.13d	5.67b	34.71e	3.77gh	23.01d	66.42	53.53b-d	1.23g
	N_4_	47.33d	1.97cd	3.43f	28.33cd	6.93f-h	4.03e	274.43d-h	47.17e-h	49.73	43.67	6.10d	5.77a	35.56e	3.97e-h	24.52d	68.57	53.77b-d	1.33g
	N_5_	89.20ab	2.70ab	5.67a-c	42.33ab	8.93a-e	5.47a-e	332.68a-d	67.00a-c	52.60	51.33	16.57ab	8.63a	143.22a-c	5.67a-c	94.05ab	65.61	67.13a	6.31a-d
P_3_	N_1_	76.83a-c	2.50a-c	5.67a-c	33.00bc	8.87b-e	5.30a-e	342.52ab	59.97a-e	52.33	50.67	16.10ab	8.47b	136.57a-d	5.40a-e	87.14a-c	63.68	66.30a	5.74b-e
	N_2_	90.63a	2.80a	6.13a	47.67a	10.40ab	6.07ab	357.33a	71.10a	54.93	51.43	17.50a	8.73b	152.94ab	5.73a-c	100.44a	65.57	67.47a	6.74ab
	N_3_	47.63d	1.97cd	3.67d-f	28.33cd	6.47gh	4.30c-e	306.13a-f	47.30e-h	48.37	44.57	6.10d	5.77a	34.94e	3.97e-h	23.96d	68.67	53.87b-d	1.29g
	N_4_	46.13d	1.90cd	3.57d-f	27.20cd	6.27gh	4.27de	225.03h	44.33f-h	47.77	44.43	5.90d	5.63a	33.42e	3.83f-h	22.80d	67.80	53.13cd	1.23g
	N_5_	88.77ab	2.77a	5.47a-d	46.00a	9.53a-d	5.73a-e	299.61a-g	69.77ab	53.30	51.10	17.13a	8.53b	146.41a-c	5.57a-d	95.57ab	65.14	67.83a	6.45a-c
P_4_	N_1_	73.23a-c	2.47a-c	4.57a-f	27.67cd	7.87d-g	5.03a-e	318.56a-e	57.17b-f	47.93	50.10	15.67a-c	8.17b	127.85a-d	5.17a-g	80.85a-c	63.20	63.73a-c	5.15d-f
	N_2_	89.20ab	2.77a	5.90ab	46.33a	10.40ab	6.23a	344.00ab	69.97ab	55.03	51.30	17.33a	8.93a	154.75a	5.87ab	101.62a	65.59	67.87a	6.92ab
	N_3_	47.30d	1.93cd	3.87c-f	26.67cd	6.93f-h	4.43b-e	251.63f-h	44.97f-h	48.30	43.77	6.23d	5.83b	36.45e	4.17d-h	26.08d	71.29	50.20d	1.31g
	N_4_	47.60d	1.93cd	4.00b-f	26.33cd	7.47e-h	4.40b-e	245.97gh	44.20f-h	49.27	44.57	6.00d	5.83b	35.12e	4.13d-h	24.92d	70.74	50.83d	1.28g
	N_5_	86.83ab	2.77a	5.10a-f	43.67ab	9.67a-c	5.30a-e	341.50a-c	68.73a-c	49.17	51.67	17.20a	8.57a	147.44a-c	5.53a-d	95.24ab	64.55	67.57a	6.42a-c
P_5_	N_1_	71.97bc	2.43a-d	4.43a-f	27.00cd	8.87b-e	4.97a-e	313.64a-e	56.17c-g	49.83	50.47	15.53a-c	8.20b	127.54a-d	5.20a-g	80.94a-c	63.32	63.47a-c	5.11d-f
	N_2_	88.77ab	2.83a	6.10a	47.67a	10.67a	6.00a-c	339.22a-c	69.70ab	50.63	50.33	17.33a	8.47a	146.65a-c	5.47a-d	94.65ab	64.50	68.00a	6.44a-c
	N_3_	47.10d	1.93cd	3.67d-f	26.33cd	6.43gh	4.23de	248.70f-h	44.43f-h	47.70	45.33	5.77d	5.80b	33.36e	4.43b-h	25.43d	76.57	50.30d	1.28g
	N_4_	46.27d	1.93cd	3.67d-f	26.00cd	6.93f-h	4.27de	245.58gh	43.30gh	48.80	43.77	6.00d	6.27b	37.57e	4.40c-h	26.42d	70.11	51.20d	1.35g
	N_5_	88.57ab	2.77a	5.43a-e	42.67ab	10.00a-c	5.83a-d	353.63a	69.43a-c	54.10	51.33	17.13a	8.60a	147.51a-c	5.70a-c	97.88a	66.14	67.33a	6.56ab
CV		8.25	8.23	13.51	10.72	6.79	10.71	6.22	7.54	7.49	6.21	5.82	6.51	8.94	9.50	11.25	4.34	5.72	9.62

Here, P_1_, P_2_, P_3_, P_4_ and P_5_ denote control (no PGR), GA_3_, SA, NAA and 4-CPA at 50 ppm, respectively and N_1_, N_2_, N_3_, N_4_ and N_5_ represent control (farmers’ practice), 80 %, 90 %, 100 % and 110 % of recommended fertilizer dose, respectively; CV: Coefficient of variation, PHT: Plant height (cm), BDM: Base diameter (cm), BNN: Number of branches per plant, LFN.P: Number of leaves per plant, LFT.L: Number of leaflets per leaf, INDL: Internode length (cm), SLFA: Single leaf area (cm^2^), CNPY: Canopy dimension (cm), SPAD: SPAD value, DFLR: Days to flowering, FLC.P: Number of flower clusters per plant, FLR.C: Number of flowers per cluster, FLR.P: Number of flowers per plant, FRT.C: Number of fruits per cluster, FRT.P: Number of fruits per plant, FSP: Fruit set percentage, FWT: Individual fruit weight (g), FYP: Fruit yield per plant (kg).

**Fig. 3:**
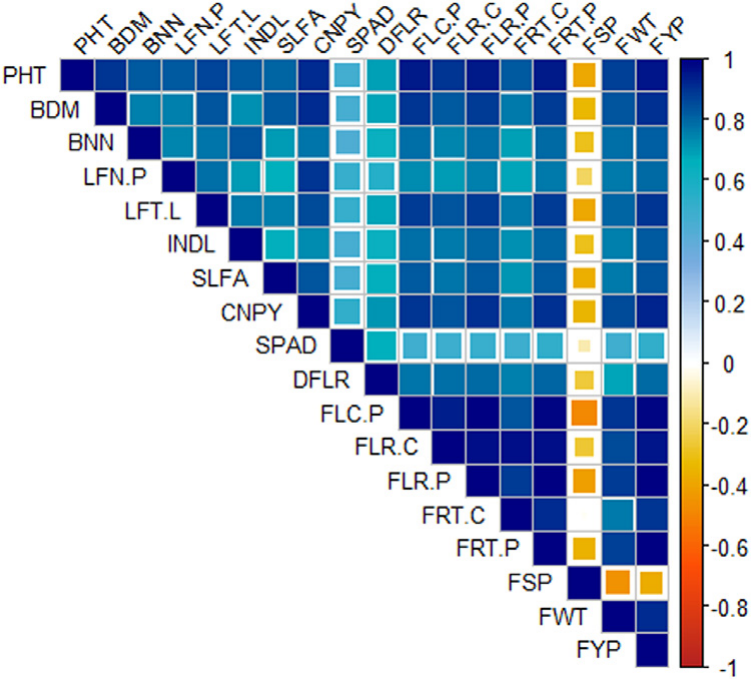
Correlation matrix exhibiting the interrelationships among the eighteen variables as a function of interactive plant growth regulator and fertilizer application in tomato. Here, PHT: Plant height (cm), BDM: Base diameter (cm), BNN: Number of branches per plant, LFN.P: Number of leaves per plant, LFT.L: Number of leaflets per leaf, INDL: Internode length (cm), SLFA: Single leaf area (cm^2^), CNPY: Canopy dimension (cm), SPAD: SPAD value, DFLR: Days to flowering, FLC.P: Number of flower clusters per plant, FLR.C: Number of flowers per cluster, FLR.P: Number of flowers per plant, FRT.C: Number of fruits per cluster, FRT.P: Number of fruits per plant, FSP: Fruit set percentage, FWT: Individual fruit weight (g), FYP: Fruit yield per plant (kg).

Again, correlation matrix showed strong to weak positive and negative correlations among the 18 studied variables as a result of interactive plant growth regulator and mineral fertilizer application in tomato (Fig. 3). Here, plant height, base diameter, number of branches and leaves per plant, internode length, canopy dimension showed moderate to very strong positive correlations with the reproductive and yield traits, specifically the number of flowers and fruits per plant, single fruit weight and yield. This implies that the conjugal utilization of PGRs and fertilizers efficiently enhanced tomato plant growth, resulting in greater yield and related features.

**Fig. 4:**
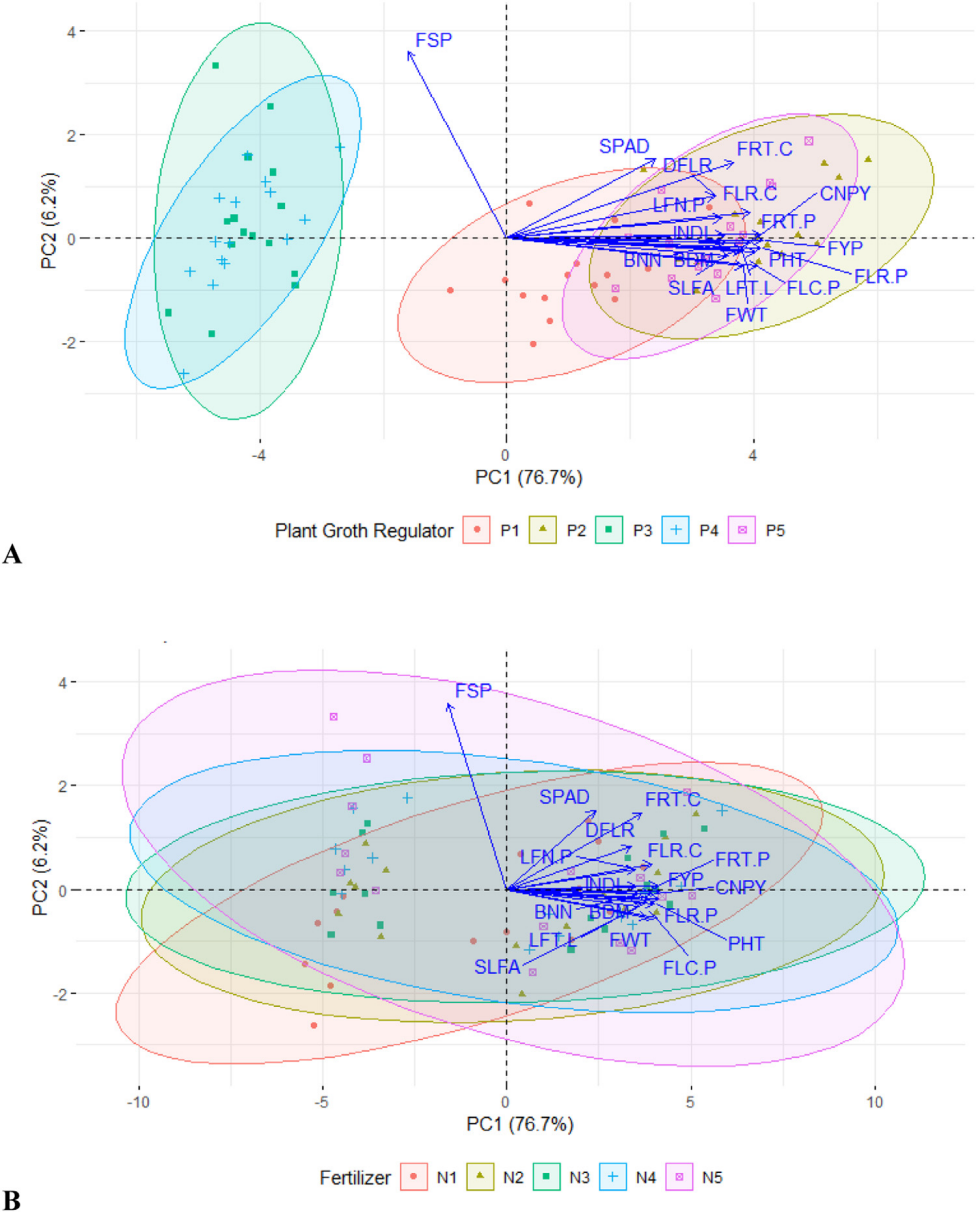
Principal component analysis (PCA) explaining the relative degree of influence on growth and yield contributing traits by plant growth regulator (A) and fertilizer (B). Eclipses with varied colors denote different treatments in PCA-biplot. Here, P_1_, P_2_, P_3_, P_4_ and P_5_ denote control (no PGR), GA_3_, SA, NAA and 4-CPA at 50 ppm, respectively and N_1_, N_2_, N_3_, N_4_ and N_5_ represent control (farmers’ practice), 80 %, 90 %, 100 % and 110 % of recommended fertilizer dose, respectively; PHT: Plant height (cm), BDM: Base diameter (cm), BNN: Number of branches per plant, LFN.P: Number of leaves per plant, LFT.L: Number of leaflets per leaf, INDL: Internode length (cm), SLFA: Single leaf area (cm^2^), CNPY: Canopy dimension (cm), SPAD: SPAD value, DFLR: Days to flowering, FLC.P: Number of flower clusters per plant, FLR.C: Number of flowers per cluster, FLR.P: Number of flowers per plant, FRT.C: Number of fruits per cluster, FRT.P: Number of fruits per plant, FSP: Fruit set percentage, FWT: Individual fruit weight (g), FYP: Fruit yield per plant (kg).

However, the PCA-biplot analysis displayed that the different fertilizer treatments overlapped with each other without forming distinct separate clusters, only there was a slight disposition of 80–110 % treatments from control treatment (Fig. 4A). From Fig. 4B, the PGR treatments GA_3_ and SA took their position in close contact at the right quadrant with maximum influence on the variables. While NAA and 4-CPA had their place at the left quadrant to show negative influence on tomato production even lower than control PGR (Fig. 4B). These phenomena can be explained that GA_3_ and SA ameliorate the fertilizer rate to regulate tomato growth and yield under studied weather conditions.

## 4 Experimental Design, Materials and Methods

### 4.1 Site and planting material

The research plot was situated at the Horticulture research field at the Department of Horticulture, Bangabandhu Sheikh Mujibur Rahman Agricultural University under Madhupur Tract (Agro-ecological Zone 28) of Bangladesh. Geographically the field had its location at 24.037°N latitude and 90.398°E longitude (8.4 m elevation from the mean sea level). BARI Tomato-14 (a variety released from Bangladesh Agricultural Research Institute) was used as planting material.

### 4.2 Treatment, layout and design

The experiment was conducted in pots using a factorial randomized complete block design (RCBD) with three replications. Seedlings that were 25 days old were moved to plastic pots measuring 30 cm × 30 cm filled with cowdung, compost, soil at 2:1:4 ratio. The experimental treatments comprised of four distinct doses of fertilizers namely 110 %, 100 %, 90 %, and 80 % of the recommended rates specified in Fertilizer Recommendation Guide [6], in addition to a control group where the 100 % dose represents 12 g of urea, 10 g of triple super phosphate (TSP), 5 g of muriate of potash (MoP), 3 g of Gypsum, 0.5 g of ZnSO_4_ (GB Zinc) and 0.5 g of H_3_BO_4_ (Solubor) per plant. While four types of PGRs like Gibberellic acid (GA_3_), Naphthalene acetic acid (NAA), 4-Chlorophenoxy acetic acid (4-CPA), and Salicylic acid (SA) were utilized at a concentration of 50 ppm each besides control (no PGR). Intercultural activities, including weeding, irrigation, mulching, pest and disease management, were performed as required.

### 4.3 Parameters studied

The interactive effect of fertilizers and PGRs on the vegetative growth behaviors of tomatoes was noted by measuring the plant's height (cm), base diameter (mm), number of branches and leaves per plant, number of leaflets per leaf, single leaf area (cm^2^), internode length (cm), canopy spread (cm) and SPAD value at flowering stage. In addition, the length between nodes (cm) and the level of greenness in the leaves (as SPAD value) were assessed during full blossom. Changes in reproductive behaviors were evaluated by recording days to flowering, number of flower clusters per plant, number of flowers and fruits per cluster, total number of flowers and fruits per plant, individual fruit weight (g) and ultimately yield per plant (kg).

### 4.4 Statistical analysis and interpretation

A two-way analysis of variance was performed. Then the data were subject to mean separation test with Tukey's HSD at *p* < 0.05 to be statistically significant. Furthermore, correlation matrix was done to examine the multidimensional relations among the growth and yield related variables. Principal component analysis (PCA) was performed to find out insight into the effects of plant growth regulator and fertilizer treatments on tomato growth, reproduction and yield. Statistical package program ‘R’ (version 4.2.2) was used to analyze and visualize the data.

## Limitations

The article demonstrates the interactive influences of different fertilizer doses and plant growth regulators on vegetative and reproductive growth attributes of tomato under fluctuating late winter situations, but it deficits the data on biochemical and physiological alteration of tomato upon the use of plant growth regulators and fertilizers.

## Ethics Statement

Involvement of animal or human based treatments have been avoided to perform this research. All authors are concerned about the ethical guidelines for publication in Data in Brief. Additionally, the study fulfills all publication requirements. The authors also agreed to publish the manuscript in Data in Brief.

## CRediT Author Statement

**Joydeb Gomasta:** Conceptualization, Methodology, Investigation, Writing - Original Draft, Writing - Review & Editing, Formal analysis, Visualization, **Jahidul Hassan:** Conceptualization, Methodology, Writing - Review & Editing, Supervision, Project administration, **Hasina Sultana:** Investigation, Writing - Original Draft, Writing - Review & Editing, **Emrul Kayesh:** Formal analysis, Data Curation, Writing - Review & Editing, Sharmila Rani Mallick: Data Curation, Writing - Review & Editing.

## Data Availability

Mendeley DataInteractive plant growth regulator and fertilizer application dataset on growth and yield attributes tomato (Solanum lycopersicum L.) (Original data).

## Appendix Supplementary materials

Image, application 1

## References

[1] Viuda-Martos M., Sanchez-Zapata E., Sayas-Barberá E., Sendra E., PérezÁlvarez J.A., Fernández-López J. (2014). Tomato and tomato byproducts. Human health benefits of lycopene and its application to meat products: a review. Crit. Rev. Food Sci. Nutr..

[2] Martí R., Roselló S., Cebolla-Cornejo J. (2016). Tomato as a source of carotenoids and polyphenols targeted to cancer prevention. Cancers (Basel).

[3] Li Y., Wang H., Zhang Y., Martin C. (2018). Can the world's favorite fruit, tomato, provide an effective biosynthetic chassis for high-value metabolites?. Plant Cell Rep..

[4] FAOSTAT, FAOSTAT Production Database, (2022), Available online at: https://www.fao.org/faostat/en/#home.

[5] BBS, Yearbook of Agricultural Statistics-2023, Bangladesh Bureau of Statistics (BBS), Statistics and Informatics Division (SID), Ministry of Planning, Government of the People's Republic of Bangladesh, (2024). Available at: https://www.bbs.gov.bd

[6] S. Ahmed, M. Jahiruddin, S. Razia, R.A. Begum, J.C. Biswas, A.S.M.M. Rahman, M.M. Ali, K.M.S. Islam, M.M. Hossain, M.N. Gani, G.M.A. Hossain, M.A. Satter, Fertilizer Recommendation Guide-2018, Bangladesh Agricultural Research Council (BARC), Farmgate, Dhaka-1215, (2018) 223p.

[7] Sharma N., Singhvi R. (2017). Effects of chemical fertilizers and pesticides on human health and environment: a review. Int. J. Agricult., Environ. Biotechnol..

[8] Rahman A., Salma U., Gomasta J., Ali M.K., Abdul Bari A.K.M., Alam M.N., Rahman M.M., Promi R.J., Kayesh E. (2023). Degree and frequency of nitrogen amendments influencing the off-season okra production in the semi-arid north-western Bangladesh. Plant Arch..

[9] Sultana N., Mannan M.A., Khan S.A.K.U., Gomasta J., Roy T. (2022). Effect of different manures on growth, yield and profitability of small scale brinjal (egg-plant) cultivation in gunny bag. Asian J. Agricult. Horticult. Res..

[10] E. Kayesh, J. Gomasta, N. Bilkish, K.A. Koly, S.R. Mallick, A holistic approach of organic farming in improving the productivity and quality of horticultural crops. In *Organic Fertilizers-New Advances and Applications*, (2023) 35p. IntechOpen, 10.5772/intechopen.1001589

[11] Maqbool A., Ali S., Rizwan M., Arif M.S., Yasmeen T., Riaz M., Hussian A., Noreen S., Abdel-Daim M.M., Alkahtani S. (2020). N-fertilizer (Urea) enhances the phytoextraction of cadmium through *Solanum nigrum* L. Int. J. Environ. Res. Public Health.

[12] Gomasta J., Uddin A.M., Kayesh E., Islam M., Haque M.A., Alam A., Islam M.T. (2024). Dataset describing the influence of preharvest gibberellic acid application on fruiting behavior, yield and fruit biochemical properties of rambutan (*Nephelium lappaceum* L.). Data Br..

[13] Uddin A.S.M.M., Gomasta J., Islam M.T., Islam M., Kayesh E., Karim M.R. (2024). Gibberellic acid spray modulates fruiting, yield, quality, and shelf life of Rambutan (*Nephelium lappaceum* L.). J. Horticult. Res..

[14] Dhakal S., Hassan J., Rajib M.M.R., Ghosh T.K., Gomasta J., Biswas M.S., Ozaki Y., Shanta S.H., Rahman M.M. (2023). Seed priming and GA_3_ field application enhanced growth, yield and postharvest quality of okra. Trends Horticult..

[15] Liu J., Qiu G., Liu C., Li H., Chen X., Fu Q., Lin Y., Guo B. (2022). Salicylic acid, a multifaceted hormone, combats abiotic stresses in plants. Life.

[16] Howlader M.I.A., Gomasta J., Rahman M.M. (2019). Integrated nutrient Management for Tomato in the southern region of Bangladesh. Int. J. Innovat. Res..

[17] Hariyadi B.W., Nizak F., Nurmalasari I.R., Kogoya Y. (2019). Effect of dose and time of npk fertilizer application on the growth and yield of tomato plants (*Lycopersicum esculentum* Mill). Agricult. Sci..

[18] Kumar S., Singh R., Singh V., Singh M.K., Singh A.K. (2018). Effect of plant growth regulators on growth, flowering, yield and quality of tomato (*Solanum lycopersicum* L.). J. Pharmacogn. Phytochem..

[19] Jakhar D., Thaneshwari S.N., Jakhar N. (2018). Effect of plant growth regulator on growth, yield & quality of tomato (*Solanum lycopericum*) cultivar ‘Shivaji'under Punjab condition. Int. J. Curr. Microbiol. Appl. Sci..

