# Supplementary material for: Interactive plant growth regulator and fertilizer application dataset on growth and yield attributes of tomato (Solanum lycopersicum L.)
Source: Data Brief. 2024 Nov 14;57:111136. doi: 10.1016/j.dib.2024.111136 (PMC11617992; doi:10.1016/j.dib.2024.111136)
Supplement: Supplementary file 1 [file mmc1.docx]

**Supplementary Material**

**Supplementary Table 1.** Main effect of fertilizer and PGR treatment on growth and yield of tomato

| **Treatment** | **PHT** | **BDM** | **BNN** | **LFN.P** | **LFT.L** | **INDL** | **SLFA** | **CNPY** | **SPAD** | **DFLR** | **FLC.P** | **FLR.C** | **FLR.P** | **FRT.C** | **FRT.P** | **FSP** | **FWT** | **FYP** |
| --- | --- | --- | --- | --- | --- | --- | --- | --- | --- | --- | --- | --- | --- | --- | --- | --- | --- | --- |
| *Plant growth regulator* | | | | | | | | | | | | | | | | | | |
| P1 | 71.20b | 2.13b | 4.34 | 28.11c | 8.51c | 5.06c | 314.41b | 56.39b | 49.42bc | 50.05a | 15.24b | 8.19b | 124.96b | 5.18b | 79.02b | 64.73c | 57.47b | 3.40b |
| P2 | 87.90a | 2.37a | 4.60 | 47.13a | 10.19a | 6.17a | 337.66a | 69.29a | 53.08a | 50.68a | 16.85a | 8.80a | 148.12a | 5.79a | 97.33a | 65.74bc | 61.07a | 4.10a |
| P3 | 47.67c | 2.39a | 4.90 | 26.13c | 6.38e | 4.32d | 270.77c | 46.07c | 47.87c | 44.27b | 5.98c | 5.58c | 33.38c | 3.89c | 23.26c | 66.17a-c | 61.72a | 4.29a |
| P4 | 46.78c | 2.37a | 4.69 | 25.77c | 6.91d | 4.20d | 247.89d | 44.09c | 48.15c | 44.02b | 5.93c | 5.71c | 34.00c | 3.92c | 23.40c | 67.07ab | 60.04a | 4.21a |
| P5 | 86.61a | 2.38a | 4.66 | 43.47b | 9.36b | 5.53b | 327.75ab | 67.51a | 52.01ab | 51.35a | 16.58a | 8.57a | 142.23a | 5.59a | 92.94a | 68.13a | 60.06a | 4.15a |
| *Fertilizer dose* | | | | | | | | | | | | | | | | | | |
| N1 | 63.41b | 2.39b | 4.65c | 31.44b | 7.94b | 4.92 | 286.33b | 53.39b | 48.59 | 47.33 | 10.93b | 7.11 | 85.44b | 4.62 | 55.52b | 63.12c | 62.91b | 4.97c |
| N2 | 69.38a | 2.72a | 5.85a | 34.67a | 8.05b | 5.08 | 305.54a | 57.85a | 50.44 | 48.07 | 12.27a | 7.37 | 97.55a | 4.84 | 63.64a | 65.67b | 67.53a | 6.57a |
| N3 | 70.00a | 1.95c | 3.67d | 36.44a | 8.31ab | 5.13 | 306.13a | 58.49a | 51.34 | 48.44 | 12.55a | 7.43 | 100.86a | 4.90 | 65.98a | 69.54a | 51.35c | 1.20d |
| N4 | 68.83a | 1.91c | 3.67d | 34.13a | 8.47a | 5.08 | 300.33a | 57.01a | 49.94 | 48.28 | 12.49a | 7.47 | 100.32a | 4.97 | 65.74a | 68.29a | 51.77c | 1.22d |
| N5 | 68.53a | 2.67a | 5.36b | 33.93ab | 8.58a | 5.06 | 300.16a | 56.61a | 50.21 | 48.25 | 12.35a | 7.47 | 98.53a | 5.04 | 65.07a | 65.22bc | 66.81a | 6.19b |
| CV | 8.25 | 8.23 | 13.51 | 10.72 | 6.79 | 10.71 | 6.22 | 7.54 | 7.49 | 6.21 | 5.82 | 6.51 | 8.94 | 9.50 | 11.25 | 4.34 | 5.72 | 9.62 |

Here, P_1_, P_2_, P_3_, P_4_ and P_5_ denote control (no PGR), GA_3_, SA, NAA and 4-CPA at 50 ppm, respectively and N_1_, N_2_, N_3_, N_4_ and N_5_ represent control (farmers’ practice), 80%, 90%, 100% and 110% of recommended fertilizer dose, respectively; PHT: Plant height (cm), BDM: Base diameter (cm), BNN: Number of branches per plant, LFN.P: Number of leaves per plant, LFT.L: Number of leaflets per leaf, INDL: Internode length (cm), SLFA: Single leaf area (cm^2^), CNPY: Canopy dimension (cm), SPAD: SPAD value, DFLR: Days to flowering, FLC.P: Number of flower clusters per plant, FLR.C: Number of flowers per cluster, FLR.P: Number of flowers per plant, FRT.C: Number of fruits per cluster, FRT.P: Number of fruits per plant, FSP: Fruit set percentage, FWT: Individual fruit weight (g), FYP: Fruit yield per plant (kg)
